# Nosocomial methicillin-resistant *Staphylococcus aureus *(MRSA) bacteremia in Taiwan: Mortality analyses and the impact of vancomycin, MIC = 2 mg/L, by the broth microdilution method

**DOI:** 10.1186/1471-2334-10-159

**Published:** 2010-06-07

**Authors:** Jiun-Ling Wang, Jann-Tay Wang, Wang-Huei Sheng, Yee-Chun Chen, Shan-Chwen Chang

**Affiliations:** 1 Department of Internal Medicine, E-Da Hospital/I-Shou University, Kaohsiung, Taiwan; 2 Department of Internal Medicine, National Taiwan University Hospital, National Taiwan University, Taipei, Taiwan

## Abstract

**Background:**

Previous studies regarding the prognosis of patients infected with MRSA isolates characterized by a high minimum inhibitory concentration (MIC) for vancomycin have generally used a commercial Etest. Little research has been conducted on determining the vancomycin susceptibility of MRSA using a reference microdilution. Additionally, there is discordance between the MIC result from an Etest and the value determined using the reference microdilution method.

**Methods:**

Using a reference microdilution method, we determined the MIC of vancomycin for isolates from 123 consecutive patients with nosocomial MRSA bacteremia. The clinical features and outcome for these patients were recorded and the MRSA isolates were genotyped.

**Results:**

Among the 123 non-duplicated isolates, 21.1% had a MIC = 2 mg/L, 76.4% had a MIC = 1 mg/L and 2.4% had MIC = 0.5 mg/L. Patients with MRSA bacteremia in the ICU or those who had been hospitalized for a long time were more likely to be infected with strains of high vancomycin MIC MRSA (MIC = 2 mg/L; *p *< 0.05). Cox regression analysis demonstrated that the high MIC group had a significantly higher 30-day mortality than the low MIC group (HR: 2.39; 95% CI: 1.20-4.79; *p *= 0.014). Multivariate analyses indicated that the presence of high MIC isolates, pneumonia, post-cardiothoracic surgery and a high Charlson comorbidity index were all independent predictors of a 30-day mortality. Genotyping of these high vancomycin MIC isolates demonstrated that SCC*mec *III, *spa *type037, was the predominant strain (> 80%). The rates of resistance to trimethoprim/sulfamethoxazole, gentamicin, levofloxacin, rifampin and tetracycline were also higher in the high MIC group than in the isolates belonging to low MIC group (*p *< 0.05).

**Conclusions:**

In a high vancomycin MIC group in Taiwan, SCC*mec *III, *spa *type t037, was the predominant strain of MRSA identified. Patients with MRSA bacteremia in the ICU or who had prolonged hospitalization were more likely to be infected with *S. aureus *strains with high vancomycin MICs. The mortality rate was higher among patients infected with these strains compared to patients infected with low MIC strains.

## Background

In 2004, Sakoulas et al. observed that a significant risk for vancomycin treatment failure in MRSA bacteremia is first indicated by increasing vancomycin MICs that are well within the susceptible range [1]. Most of the studies examining the impact of high vancomycin MIC in patients with MRSA bacteremia published after the Sakoulas *et al*. report have used the commercial E test and found similar results [2-4]. However, a recent study, using multivariate analysis, demonstrated that there was no difference in mortality between high MIC and low MIC patient groups [5]. There were discordant MIC results between different susceptibility methods [6-8]. Furthermore, there has been little research examining the use of reference microdilution as a method of vancomycin susceptibility determination [9].

Additionally, a previous genotype study in the U.S. showed that SCC*mec *II was the genotype that was most predictive of high vancomycin MIC isolates [10]. In Taiwan, the molecular epidemiology of isolates from patients with MRSA bacteremia is distinct [11]. We do not know if the high vancomycin MIC isolates from patients in Taiwan have a similar genotype as in those seen in patients in the U.S.

Therefore, we collected bacteria from consecutive patients with nosocomial MRSA bacteremia and, using the Clinical and Laboratory Standards Institute (CLSI) reference broth microdilution, determined vancomycin MICs. We examined the association of infection with high MIC strains (MIC = 2 mg/L) and mortality and genotyped the isolates.

## Methods

This study was approved by the local institutional ethics review board (expedited review). The Institutional Review Board waived the need for informed consent from participants because the study involved very minimal risk to the subjects, did not include intentional deception and did not involve sensitive populations or topics; this waiver does not adversely affect the rights and welfare of the subjects. Throughout the one-year study period (January 1 to December 31, 2006), we collected clinical data from 123 non-duplicated, consecutive nosocomial MRSA bacteremia patients from the National Taiwan University Hospital in Taipei, Taiwan. During the study period, vancomycin and teicoplanin were the recommended antibiotics for treating MRSA bacteremia at our institutions. All patients were evaluated using a structured recording form. The clinical course of infection and the infection focus were evaluated and recorded according to information supplied by primary care physicians and medical records.

Identification of the infection focus was based on clinical, bacteriological and radiological investigations, and was defined according to criteria established by the Centers for Disease Control and Prevention [12]. We assessed patient survival rates 30 days after the diagnosis of bacteremia by follow-up at outpatient clinics or by telephone for patients who did not come to the outpatient clinics.

The vancomycin MIC of the 123 MRSA isolates was determined by broth microdilution, as described by the CLSI in 2005 [13]. In vitro testing of these isolates was performed in a blinded fashion without knowledge of any clinical outcomes. Isolates with vancomycin MIC = 2 mg/L were designated the "high MIC" group; those with MIC = 1 mg/L or 0.5 mg/L were considered to be the "low MIC" group. The susceptibility of the *S. aureus *isolates to levofloxacin, erythromycin, tetracycline, trimethoprim/sulfamethoxazole, gentamicin, clindamycin and rifampin was determined according to standard microbiological methods [13]. The presence of the SCC*mec *elements (I-V) was determined by previously described methods [14,15]. The polymorphic X-region of the protein A gene (*spa*) was analyzed as previously described [16].

Percentages were used for all categorical variables. For univariate analysis, we compared the high MIC and low MIC groups with a χ^2 ^test or Fisher's exact test. We used multivariate logistic regression analyses to determine associations between potential risk factors and the presence of isolates with a high or low vancomycin MIC. The cumulative survival time after the first MRSA positive blood culture was calculated using the Kaplan-Meier method. The difference in cumulative survival of patients infected with high or low vancomycin MIC *S. aureus *was determined using the log-rank test. The effect of high MIC isolates on patient outcome was evaluated using a multivariate Cox proportional hazards regression model and a logistic regression model adjusted for age, sex and underlying comorbidities. Data were analyzed using SPSS software for Windows (Release 15.0; SPSS, Chicago, IL).

## Results

Among the 123 non-duplicated isolates, 21.1% had a MIC = 2 mg/L, 76.4% had a MIC = 1 mg/L and 2.4% had a MIC = 0.5 mg/L (Table 1). Univariate analysis indicated that patients with MRSA bacteremia who were in the ICU (*p *= 0.028) or who had been hospitalized for a long time, i.e., > 60 days, (*p *= 0.022) were more likely to be infected with MRSA strains with a high vancomycin MIC (MIC = 2 mg/L). Multivariate analysis indicated that this effect remained after adjusting for sex, age and the presence of underlying disease. There were no other significant demographic differences between the high MIC and low MIC groups (Table 1). The infection syndrome, treatment and outcome are shown in Table 2.

**Table 1:** Demographic characteristics of nosocomial bacteremia patients infected with high MIC or low MIC MRSA

Characteristic	Vancomycin MIC < 2 N = 97 Number of patients (%)	Vancomycin MIC = 2 N = 26 Number of patients (%)	*p*-value*^a^*	*p*-value*^b^*; OR (95% CI)
Elderly (>65 years old)	66 (68.0)	13 (50.0)	0.088	
Age	70.32 ± 14.1	63.6 ± 18.0	0.250	
Sex: male	68 (70.1)	14 (53.8)	0.118	0.04; 0.33 (0.11-0.95)
Diabetes	70 (72.2)	17 (65.4)	0.500	
Cancer	30 (30.9)	11(43.2)	0.349	
Congestive heart failure	14 (14.4)	4(15.4)	1.000	
End stage renal disease	10 (10.3)	4 (15.4)	0.492	
Liver cirrhosis	11 (11.3)	3 (11.5)	1.000	
Cerebrovascular disease	24 (24.7)	3 (11.5)	0.188	
Bedridden	24 (24.7)	7 (26.9)	0.804	
Recent surgery	24 (24.7)	3 (11.5)	0.188	0.018; 0.15 (0.03-0.73)
Recent cardio-thoracic surgery	13 (13.4)	1 (3.8)	0.297	
Hospitalization days before bacteremia	22.6 ± 10.1	23.7 ± 8.0	0.207	
ICU admission before bacteremia	40 (41.2)	17 (65.4)	0.028	0.005; 4.83 (1.61-14.50)
Hospitalization > 2 months before bactermia	17 (17.5)	10 (38.5)	0.022	0.011; 4.56 (1.41-14.73)
Hospitalization > 1 month before bacteremia	41 (42.3)	15 (57.7)	0.187	

*a: p-*value calculated using a χ^2 ^test or Fisher's exact test

*b*: *p*-value as determined by logistic regression.

**Table 2:** Infection syndrome, treatment and outcome of nosocomial bacteremia in patients infected with high MIC MRSA or low MIC MRSA

Syndrome & treatment	Vancomycin MIC < 2 N = 97 Number of patients (%)	Vancomycin MIC = 2 N = 26 Number of patients (%)	*p-*value*^a^*
**Syndrome**			
Primary bacteremia	18 (18.6)	5 (19.2)	0.938
Pneumonia	32 (33.0)	9 (34.6)	0.876
Catheter related infection	40 (41.2)	12 (46.2)	0.652
Prosthesis	6(6.2)	0 (0)	0.341
Infective endocarditis	6 (6.2)	0 (0)	0.341
**Treatment**			
Empirical glycopeptide use within 48 hours	71 (73.2)	19 (73.1)	1.000
Vancomycin trough level (mg/L)	14.9 ± 7.6 (n = 47)	13.5 ± 4.9 (n = 12)	0.176
Vancomycin trough level > 10 mg/L in first week	34 (72.3)	9 (75.0)	1.000
With a DNR order^b^	26 (26.8)	11 (42.3)	0.151
**Outcome**			
2 week death	14 (14.4)	8 (30.8)	0.081
30 days death	27 (27.8)	13 (50.0)	0.057

*a: p-*value calculated using a χ^2 ^test or Fisher's exact test

*b: *Do not resuscitate

**Figure 1:**
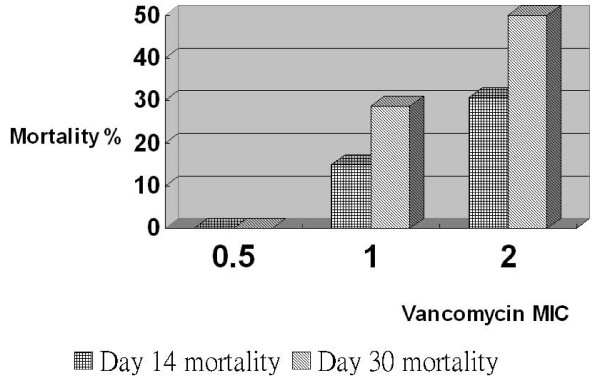
**Mortality in different vancomycin MIC group**. The relationship between vancomycin MIC and 14- and 30-days mortality

The relationship between MIC and Day 14 and Day 30 mortality is shown in Figure 1. Additionally, we used Cox regression and logistic regression to evaluate the relationship between vancomycin MIC and mortality. Univariate analyses indicated that the presence of high MIC isolates, malignancy, a high Charlson comorbidity index, bedridden status and admission to the ICU were predictors of mortality after 30 days (*p *< 0.05) (Table 3). Variables with a *p*-value < 0.20 in the univariate analyses were included in the subsequent multivariate Cox proportional hazards regression model. Multivariate analyses in combination with Cox regression indicated that the presence of high MIC isolates, pneumonia, history of cardiothoracic surgery and a high Charlson comorbidity index were independent predictors of mortality after 30 days (*p *< 0.05). Patients infected with high MIC isolates had a greater 30-day mortality rate than patients infected with low MIC isolates, as indicated by univariate analysis (hazard ratio (HR) = 2.20; 95% confidence interval (CI): 1.13-4.27; *p *= 0.021) and multivariate Cox regression analysis (HR = 2.39; 95% CI: 1.20-4.79; *p *= 0.014). By using stepwise logistic regression, the presence of high MIC isolates, cancer, a high Charlson comorbidity index and a history of cardiothoracic surgery were shown to be independent predictors of mortality after 30 days (*p *< 0.05) (Table 4).

**Table 3:** Univariate and multivariate analysis of risk factors associated with mortality of patients with nosocomial MRSA bacteremia using Cox regression

Characteristic	Univariate HR	95% CI	*p*-value	Multivariate HR	95% CI	*p*-value
High vancomycin MIC	2.20	1.13-4.27	0.021	2.39	1.20-4.79	0.014
Malignancy	2.73	1.45-5.11	0.002	2.09	0.99-4.43	0.053
Pneumonia	1.74	0.92-3.27	0.088	2.27	1.17-4.40	0.016
Cardiothoracic surgery	1.86	0.82-4.20	0.139	4.38	1.74-11.04	0.002
Charlson score	1.27	1.10-1.46	0.001	1.24	1.07-1.45	0.005
Endocarditis	2.24	2.24-6.31	0.126			
Bedridden	2.63	1.03-6.71	0.044			
Cerebrovascular disease	2.06	0.81-5.27	0.131			
ICU admission	2.05	1.07-3.90	0.029			

Note: Variables with a *p *< 0.2 in the univariate analyses were included in the subsequent multivariate Cox proportional hazards regression model.

**Table 4:** Univariate and multivariate analyses of risk factors associated with mortality in patients with nosocomial MRSA bacteremia using logistic regression

Characteristic	Univariate OR	95% CI	*p*-value	Multivariate OR*^a^*	95% CI	*p*-value
High vancomycin MIC	2.59	1.07-6.30	0.035	3.76	1.34-10.54	0.012
Malignancy	3.48	1.57-7.74	0.002	3.06	1.15-8.17	0.025
Pneumonia	1.82	0.83-3.99	0.137			
Cardiothoracic surgery	2.30	0.75-7.09	0.146	5.66	1.52-21.12	0.010
Charlson score	1.27	1.06-1.52	0.009	1.25	1.01-1.55	0.045
Endocarditis	4.50	0.79-25.69	0.09			
Bedridden	3.19	1.12-9.08	0.03			
Cerebrovascular disease	2.52	0.88-7.26	.086			
ICU admission	2.27	1.05-4.91	.037			

*a*: Covariates having a *p *< 0.2 were included in the stepwise regression, and covariates with a higher *p-*value were discarded.

**Figure 2:**
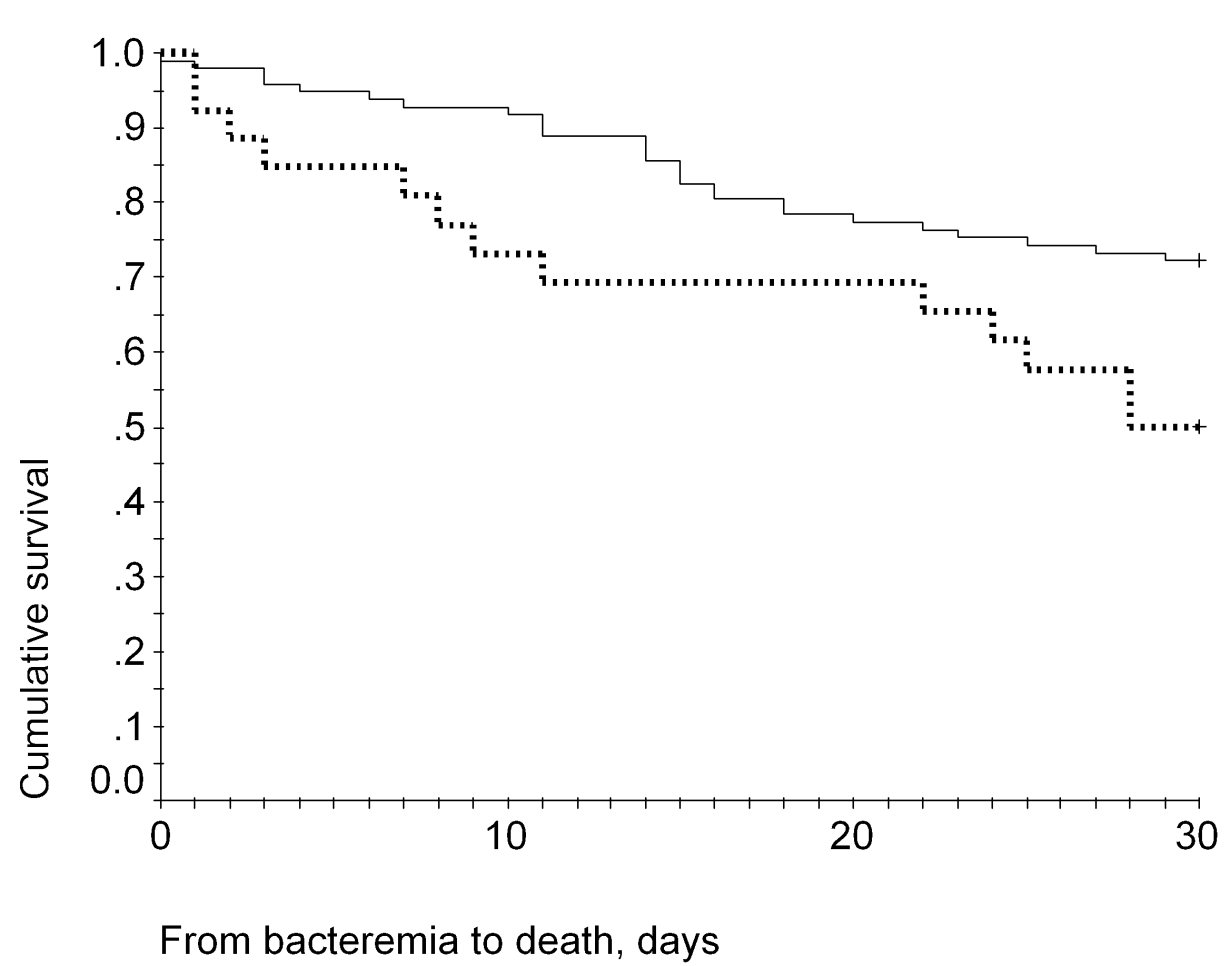
**Kaplan-Meier survival curves of high-MIC and low-MIC group**. Kaplan-Meier survival curves of patients with nosocomial MRSA bacteremia who were infected with high-MIC isolates (lower line) or low-MIC isolates (upper line). Log-rank test: *p = *0.0232

**Figure 3:**
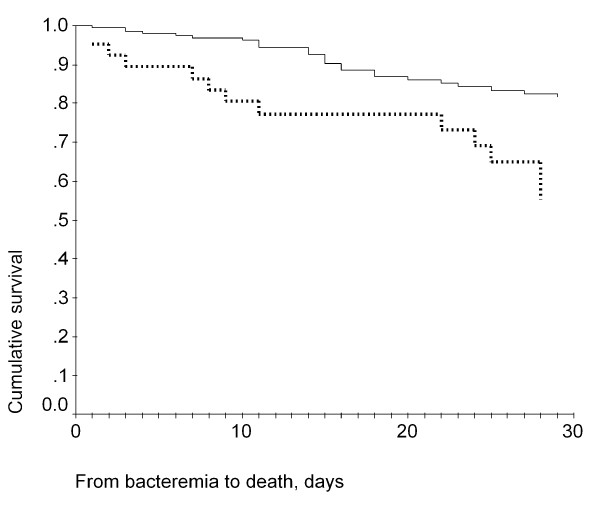
**Cox regression survival curves of high-MIC and low-MIC group**. Cox regression survival curves of patients with nosocomial MRSA bacteremia who were infected with high-MIC isolates (lower line) or low-MIC isolates (upper line)

The 30-day cumulative survival was 72.2% for patients infected with low MIC isolates and was 50% for patients infected with high MIC isolates (Figure 2). A log-rank test indicated that this difference was statistically significant (*p = 0*.0232). The Cox regression survival curves for patients infected with high MIC or low MIC isolates are shown in Figure 3. Analysis of the distribution of the SCC*mec *genotype and *spa *genotype in the high and low MIC groups indicated that most of the high-MIC isolates had the genotype SCC*mec *III, *spa *type t037 (Table 5). The high MIC group also displayed a higher resistance rate to other antibiotics (Table 5).

**Table 5:** Molecular and antimicrobial resistance features of high MIC and low MIC MRSA isolates

Characteristic	Vancomycin MIC < 2 N = 97 Number of patients (%)	Vancomycin MIC = 2 N = 26 Number of patients (%)	*p*-value*^a^*
SCC*mec *type			
*SCCmec *II	24 (24.7)	2 (7.7)	
SCC*mec *III	52 (53.6)	24 (92.3)	
SCC*mec *IV	10 (10.3)		
SCC*mec *V	11 (11.3)		
*Spa *type			
*Spa *t037	50 (51.5)	22 (84.6)	
*Spa *t002	24 (24.7)	2 (7.7)	
*Spa *t437	18 (18.6)	0 (0)	
*Spa *t298	1 (1)	0 (0)	
*Spa *t1081	1 (1)	0 (0)	
*Spa *t138	0 (0)	1 (3.8)	
Non-typeable	3 (3.1)	1 (3.8)	
**Isolates Susceptibility rate**			
Erythromycin	2 (2.1)	(0)	0.460
Clindamycin	14 (14.4)	1 (3.8)	0.143
Tetracycline	33 (34)	2 (7.7)	0.008
Levofloxacin	20 (20.6)	0 (0)	0.011
TMP/SMZ	45 (46.4)	2 (7.7)	< 0.001
Gentamicin	17 (13.4)	0 (0)	0.021
Rifampin	78 (80.4)	16 (61.5)	0.044

*a: p-*value calculated using a χ^2^-test

## Discussion

Our results show that patients infected with isolates of MRSA with a high vancomycin MIC, as determined by standard broth microdilution methods, tend to have higher mortality. This is in agreement with previous studies using Etest as the method of susceptibility determination, which displayed differing success rates (23-45%) with vancomycin treatment of patients infected with high MIC and low MIC isolates [1-5,9]. Our survival analysis indicated that patients infected with high MIC isolates had a 16% higher mortality after 14 days and a 22% higher mortality after 30 days compared to patients infected with low MIC isolates. The poor clinical outcome of patients infected with high MIC isolates remained after adjusting for confounding variables.

Contrary to the results reported by Musta et al., our study showed patients infected with MRSA with high vancomycin MIC had a higher mortality rate, as assessed by multivariate analyses, compared to patients infected with low vancomycin MIC isolates. The reason for these differences may be explained by the use of a different MIC test (Etest vs. microdilution) and different SCC*mec *genotypes, i.e., SCC*mec *III, t037. In a previous report, vancomycin MICs generated using the Etest were consistently a one- to two-fold dilution higher than MICs determined using the CLSI broth dilution method [6,7]. We think our cohort contained more patients infected with high vancomycin MIC isolates than the Musta et al. cohort; thus, their observed difference may have been more significant if their study had involved more patients infected with high vancomycin MIC *S. aureus*.

The higher mortality rate of patients infected with high MIC isolates may be explained by an inadequate AUC/MIC ratio, delayed bacterial killing in the high MIC group [1-5,9], and/or the existence of strains of heteroresistant vancomycin-intermediate *S. aureus *[17]. The higher mortality rate in high MIC group may also be associated with other covariates that were not measured in this study such as severity of acute illness (i.e.APACHE II score) or do not resuscitate orders.

In our isolates from the high MIC group, the most common (> 80%) genotype was SCC*mec *III, *spa *type t037. MRSA sequence type (ST) 239, *spa *type t037, SCC*mec *III, is now the most prevalent nosocomial strain in many Asian countries [11,18-20] and has also been detected in 26 countries outside of Asia [21]. In a study from Singapore and Hong Kong, MRSA ST239 (SCC*mec *III/*spa *type t037) was found to comprise a higher proportion of the high vancomycin MIC isolates than other SCC*mec *type strains [22,23]. The distribution of staphylococcal cassette chromosome mec types and correlation with comorbidity and infection type in our patients with MRSA bacteremia has been described in another study [24]. The high vancomycin MIC and the high resistance rate to other antibiotics associated with this major clone is a therapeutic challenge for clinicians.

Broth microdilution is a standard CLSI method for determining MICs, but this technique is time consuming and labour intensive. The limitation of this study is that it is a retrospective, single center study in Taiwan and the local MRSA vancomycin MIC distribution pattern in Taiwan may not be applicable to other countries. Clonal transmission among these MRSA isolates is also possible. Additionally, other confounding factors for the mortality analysis such as more do not resuscitate order in high MIC group than low MIC group may also exist.

## Conclusions

In conclusion, using a microdilution method, our study shows that patients with MRSA bacteremia who are in the ICU or who have been hospitalized for a long time may be infected with strains of high vancomycin MICs (MIC = 2 mg/L ). Patients infected with high-MIC strains had higher mortality than patients infected with low-MIC strains.

## Competing interests

The authors declare that they have no competing interests.

## Authors' contributions

Conceived and designed the experiments: WJL and WJT; performed the experiments: WJL and WJT; analyzed the data: WJL and WJT; wrote the paper: WJL, WJT and SWH; oversaw the running of the project: CYC and CSC. All authors have read and approved the final manuscript

## Pre-publication history

The pre-publication history for this paper can be accessed here:

http://www.biomedcentral.com/1471-2334/10/159/prepub

## References

[B1] Sakoulas G Moise-Broder PA Schentag J Forrest A Moellering RC Jr Eliopoulos GM Relationship of MIC and bactericidal activity to efficacy of vancomycin for treatment of methicillin-resistant Staphylococcus aureus bacteremia J Clin Microbiol 2004 42 2398 2402 10.1128/JCM.42.6.2398-2402.2004 15184410 PMC427878

[B2] Hidayat LK Hsu DL Quist R Shriner KA Wong-Beringer A High dose vancomycin therapy for methicillin-resistant Staphylococcusaureus infections: efficacy and toxicity Arch Intern Med 2006 166 2138 2144 10.1001/archinte.166.19.2138 17060545

[B3] Soriano A Marco F Martinez JA Pisos E Almela M Dimova VP Alamo D Ortega M Lopez J Mensa J Influence of vancomycin minimum inhibitory concentration on the treatment of methicillin-resistant Staphylococcus aureus bacteremia Clin Infect Dis 2008 46 193 200 10.1086/524667 18171250

[B4] Lodise TP Graves J Evans A Graffunder E Helmecke M Lomaestro BM Stellrecht K Relationship between vancomycin MIC and failure among patients with methicillin-resistant Staphylococcus aureus bacteremia treated with vancomycin Antimicrob Agents Chemother 2008 52 3315 3320 10.1128/AAC.00113-08 18591266 PMC2533486

[B5] Musta AC Riederer K Shemes S Chase P Jose J Johnson LB Khatib R Vancomycin MIC plus heteroresistance and outcome of methicillin-resistant Staphylococcus aureus bacteremia: trends over 11 years J Clin Microbiol 2009 47 1640 1644 10.1128/JCM.02135-08 19369444 PMC2691078

[B6] Prakash V Lewisnd JS Jorgensen JH Vancomycin MICs for methicillin-resistant Staphylococcus aureus isolates differ based upon the susceptibility test method used Antimicrob Agents Chemother 2008 52 4528 10.1128/AAC.00904-08 18838599 PMC2592869

[B7] Hsu DI Hidayat LK Quist R Hindler J Karlsson A Yusof A Wong-Beringer A Comparison of method-specific vancomycin minimum inhibitory concentration values and their predictability for treatment outcome of methicillin-resistant Staphylococcus aureus (MRSA) infections Int J Antimicrob Agents 2008 32 378 385 10.1016/j.ijantimicag.2008.05.007 18701261

[B8] Chua T Moore CL Perri MB Donabedian SM Masch W Vager D Davis SL Lulek K Zimnicki B Zervos MJ Molecular epidemiology of methicillin-resistant Staphylococcus aureus bloodstream isolates in urban Detroit J Clin Microbiol 2008 46 2345 2352 10.1128/JCM.00154-08 18508934 PMC2446903

[B9] Moise PA Sakoulas G Forrest A Schenag JJ Vancomycin in vitro bactericidal activity and its relationship to efficacy in clearance of methicillin-resistant Staphylococcus aureus bacteremia Antimicrob Agents Chemother 2007 57 2582 2586 10.1128/AAC.00939-06 PMC1913284 17452488

[B10] Moise PA Smyth DS Robinson DA El-Fawal N McCalla C Sakoulas G Genotypic and phenotypic relationships among methicillin-resistant Staphylococcus aureus from three multicentre bacteraemia studies J Antimicrob Chemother 2009 63 873 876 10.1093/jac/dkp047 19261624 PMC2667134

[B11] Wang JL Wang JT Chen SY Hsueh PR Kung HC Chen YC Chang SC Adult methicillin-resistant Staphylococcus aureus bacteremia in Taiwan: clinical significance of non-multi-resistant antibiogram and Panton-Valentine leukocidin gene Diagn Microbiol Infect Dis 2007 59 365 371 10.1016/j.diagmicrobio.2007.06.021 17878063

[B12] Garner JS Jarvis WR Emori TG Horan TC Hughes JM CDC definitions for nosocomial infections,1988 Am J Infect Control 1988 16 128 140 10.1016/0196-6553(88)90053-3 2841893

[B13] National Committee for Clinical Laboratory Standards Performance standards for antimicrobial susceptibility testing: 14th informational supplement M100-S15 NCCLS 2005 Wayne, PA

[B14] Ito T Katayama Y Asada K Mori N Tsutsumimoto K Tiensasitorn C Hiramatsu K Structural comparison of three types of staphylococcal cassette chromosome *mec *integrated in the chromosome in methicillin-resistant *Staphylococcus aureus* Antimicrob Agents Chemother 2001 45 1323 1336 10.1128/AAC.45.5.1323-1336.2001 11302791 PMC90469

[B15] Ito T Ma XX Takeuchi F Okuma K Yuzawa H Hiramatsu K Novel type V staphylococcal cassette chromosome mec driven by a novel cassette chromosome recombinase, ccrC Antimicrob Agents Chemother 2004 48 2637 2651 10.1128/AAC.48.7.2637-2651.2004 15215121 PMC434217

[B16] Harmsen D Claus H Witte W Rothganger J Claus H Turnwald D Vogel U Typing of methicillin-resistant Staphylococcus aureus in a university hospital setting by using novel software for *spa *repeat determination and database management J Clin Microbiol 2003 41 5442 5448 10.1128/JCM.41.12.5442-5448.2003 14662923 PMC309029

[B17] Charles PG Ward PB Johnson PD Howden BP Grayson ML Clinical features associated with bacteremia due to heterogeneous vancomycin-intermediate Staphylococcus aureus Clin Infect Dis 2004 38 448 451 10.1086/381093 14727222

[B18] Chongtrakool P Ito T Ma XX Kondo Y Trakulsomboon S Tiensasitorn C Jamklang M Chavalit T Song JH Hiramatsu K Staphylococcal cassette chromosome mec (SCCmec) typing of methicillin-resistant Staphylococcus aureus strains isolated in 11 Asian countries: a proposal for a new nomenclature for SCCmec elements Antimicrob Agents Chemother 2006 50 1001 1012 10.1128/AAC.50.3.1001-1012.2006 16495263 PMC1426434

[B19] Neela V Ghasemzadeh Moghaddam H van Belkum A Horst-Kreft D Mariana NS Ghaznavi Rad E First report on methicillin-resistant Staphylococcus aureus of Spa type T037, Sequence type 239, SCCmec type III/IIIA in Malaysia Eur J Clin Microbiol Infect Dis 2009 29 115 117 10.1007/s10096-009-0813-6 19779745 PMC2797423

[B20] Liu Y Wang H Du N Shen E Chen H Niu J Ye H Chen M Molecular evidence for spread of two major methicillin-resistant Staphylococcus aureus clones with a unique geographic distribution in Chinese hospitals Antimicrob Agents Chemother 2009 53 512 518 10.1128/AAC.00804-08 19029328 PMC2630620

[B21] Feil EJ Nickerson EK Chantratita N Wuthiekanun V Srisomang P Cousins R Pan W Zhang G Xu B Day NP Peacock SJ Rapid detection of the pandemic methicillin-resistant Staphylococcus aureus clone ST 239, a dominant strain in Asian hospitals J Clin Microbiol 2008 46 1520 1522 10.1128/JCM.02238-07 18234867 PMC2292922

[B22] Hsu LY Loomba-Chlebicka N Koh YL Tan TY Krishnan P Lin RT Tee NW Fisher DA Koh TH Evolving EMRSA-15 epidemic in Singapore hospitals J Med Microbiol 2007 56 376 379 10.1099/jmm.0.46950-0 17314369

[B23] Ho PL Lo PY Chow KH Lau EH Lai EL Cheng VC Kao RY Vancomycin MIC creep in MRSA isolates from 1997 to 2008 in a healthcare region in Hong Kong J Infect 2010 60 140 145 10.1016/j.jinf.2009.11.011 19961873

[B24] Wang JL Wang JT Chen SY Chen YC Chang SC Distribution of staphylococcal cassette chromosome mec Types and correlation with comorbidity and infection type in patients with MRSA bacteremia PLoS One 2010 5 e9489 10.1371/journal.pone.0009489 20221428 PMC2832693

